# Inhibition of host 5-lipoxygenase reduces overexuberant inflammatory responses and mortality associated with *Cryptococcus* meningoencephalitis

**DOI:** 10.1128/mbio.01483-24

**Published:** 2024-07-31

**Authors:** Natalia Castro-Lopez, Althea Campuzano, Elysa Mdalel, Difernando Vanegas, Ashok Chaturvedi, Phung Nguyen, Mark Pulse, Astrid E. Cardona, Floyd L. Wormley

**Affiliations:** 1 Department of Biology, Texas Christian University, Fort Worth, Texas, USA; 2 South Texas Center for Emerging Infectious Diseases, The University of Texas at San Antonio, San Antonio, Texas, USA; 3 Department of Molecular Microbiology and Immunology, The University of Texas at San Antonio, San Antonio, Texas, USA; 4 Department of Pharmacology, University of North Texas Health Science Center, Fort Worth, Texas, USA; Duke University Hospital, Durham, North Carolina, USA

**Keywords:** *Cryptococcus*, *Cryptococcosis*, 5-lipoxygenase, meningitis, meningoencephalitis, *Cryptococcus neoformans*, *Cryptococcus deneoformans*, medical mycology, fungal immunology, immunity, host–pathogen interactions, microbial pathogenesis

## Abstract

Cryptococcosis, caused by fungi of the genus *Cryptococcus*, manifests in a broad range of clinical presentations, including severe pneumonia and disease of the central nervous system (CNS) and other tissues (bone and skin). Immune deficiency or development of overexuberant inflammatory responses can result in increased susceptibility or host damage, respectively, during fungal encounters. Leukotrienes help regulate inflammatory responses against fungal infections. Nevertheless, studies showed that *Cryptococcus* exploits host 5-lipoxygenase (5-LO), an enzyme central to the metabolism of arachidonic acid into leukotrienes, to facilitate transmigration across the brain–blood barrier. To investigate the impact of host 5-LO on the development of protective host immune responses and mortality during cryptococcosis, wild-type (C57BL/6) and 5-lipoxygenase-deficient (5-LO^−/−^) mice were given experimental pulmonary and systemic *Cryptococcus* sp., infections. Our results showed that 5-LO^−/−^ mice exhibited reduced pathology and better disease outcomes (i.e., no mortality or signs associated with cryptococcal meningoencephalitis) following pulmonary infection with *C. deneoformans,* despite having detectable yeast in the brain tissues. In contrast, C57BL/6 mice exhibited classical signs associated with cryptococcal meningoencephalitis. Additionally, brain tissues of 5-LO^−/−^ mice exhibited lower levels of cytokines (CCL2 and CCL3) clinically associated with *Cryptococcus*-related immune reconstitution inflammatory syndrome (C-IRIS). In a systemic mouse model of cryptococcosis, 5-LO^−/−^ mice and those treated with a Federal Drug Administration (FDA)-approved 5-LO synthesis inhibitor, zileuton, displayed significantly reduced mortality compared to C57BL/6 infected mice. These results suggest that therapeutics designed to inhibit host 5-LO signaling could reduce disease pathology and mortality associated with cryptococcal meningoencephalitis.

**IMPORTANCE:**

Cryptococcosis is a mycosis with worldwide distribution and has a broad range of clinical manifestations, including diseases of the CNS. Globally, there is an estimated 179,000 cases of cryptococcal meningitis, resulting in approximately 112,000 fatalities per annum and 19% of AIDS-related deaths. Understanding how host immune responses are modulated during cryptococcosis is central to mitigating the morbidity and mortality associated with cryptococcosis. Leukotrienes (LTs) have been shown to modulate inflammatory responses during infection. In this study, we show that mice deficient in 5-lipoxygenase (5-LO), an enzyme central to the metabolism of arachidonic acid into leukotrienes, exhibit reduced pathology, disease, and neurological signs associated with cryptococcal meningitis. Additionally, mice given an experimental cryptococcal infection and subsequently treated with an FDA-approved 5-LO synthesis inhibitor exhibited significantly reduced mortality rates. These results suggest that therapeutics designed to inhibit host 5-LO activity could significantly reduce pathology and mortality rates associated with cryptococcal meningitis.

## INTRODUCTION

Pathogenic members of the *Cryptococcus* species*,* etiological agents of cryptococcosis, can cause severe pneumonia and diseases of the central nervous system (CNS) and other tissues (bone and skin). *Cryptococcus* sp. has a worldwide distribution and can cause diseases in immunocompetent individuals but is especially problematic in immunocompromised populations, particularly AIDS patients and those undergoing immunosuppressive therapies (reviewed in reference 1). Cryptococcal meningoencephalitis is the most commonly transmitted fungal disease in AIDS patients and is responsible for 19% of AIDS-related deaths globally (2). Cryptococcosis also accounts for 7%–8% of the invasive fungal diseases in solid organ transplant (SOT) recipients (3).

Leukotrienes (LTs) are part of a group of lipids formed by oxidizing polyunsaturated fatty acids (4, 5). 5-lipoxygenase (5-LO) metabolizes arachidonic acid into leukotrienes that play an essential role in acute and chronic inflammation and allergic disease (6). Leukotriene B_4_ (LTB_4_), one of the most common leukotrienes, is synthesized by phagocytes during inflammation or the immune response to infection (6). LTB_4_ stimulates the migration of leukocytes to sites of inflammation; activation of macrophages, neutrophils, and eosinophils (7, 8); and enhances phagocytosis and phagocyte antimicrobial activity (9, 10). Deficiency in the production of LTs is associated with impaired phagocytosis and killing of microorganisms (11, 12).

Leukotrienes help regulate the inflammatory response to infection, including the immune response to fungal infections. Blockage of leukotriene production with MK886, an inhibitor of 5-LO, in mice given an experimental infection with *Histoplasma capsulatum* results in an increase in fungal burden and inflammatory cytokine levels (8, 13, 14). 5-LO-deficient (5-LO^−/−^) mice show increased susceptibility to experimental infection with *H. capsulatum* or *Paracoccidioides brasiliensis* exhibited by increased pulmonary fungal burden and decreased phagocytosis and antimicrobial killing by macrophages, suggesting that LTs are necessary for the induction of innate immune responses and control of fungal infections (14, 15). Leukotriene receptor LTB_4_R1-deficient mice exhibit a decreased recruitment of neutrophils and eosinophils to *Aspergillus fumigatus* infection and increased susceptibility to invasive pulmonary aspergillosis (16). Previous studies show that blockage of host-derived 5-LO decreases the ability of *Cryptococcus* sp. to cross the brain–blood barrier (BBB) (17).

In the present study, we sought to determine the impact of host-derived 5-LO during the host immune response to cryptococcosis. Our data show that mice deficient in 5-LO signaling do not develop the overexuberant inflammatory responses and classical signs associated with cryptococcal meningoencephalitis, despite the presence of yeast in brain tissues. Additionally, mice treated with an FDA-approved therapeutic agent, zileuton, a 5-LO synthesis inhibitor, exhibited significantly reduced mortality rates compared to control-treated mice. Altogether, our studies suggest that inhibition of 5-LO signaling significantly reduces disease pathology and mortality rates associated with cryptococcal meningitis.

## RESULTS

### Host 5-lipoxygenase is not necessary to control dissemination of *Cryptococcus* yeast to the brain

**Fig 1:**
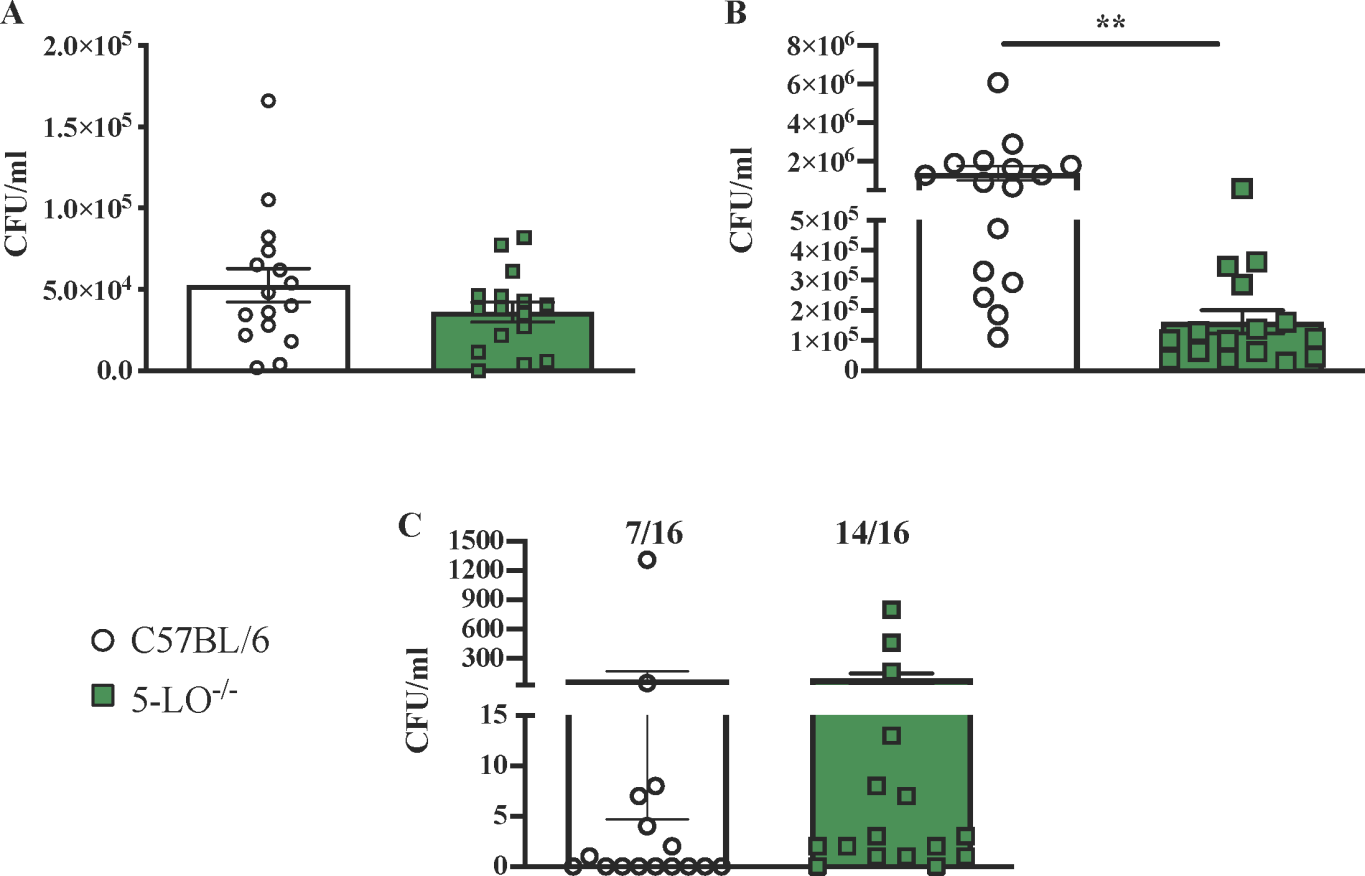
5-LO is not essential for early control of fungal burden and anti-cryptococcal activity during chronic infection with *C. deneoformans*. (A–C) C57BL/6 and 5-LO^−/−^ mice were infected with 10^4^ CFU of *C. deneoformans* strain 52D via intranasal inhalation. Pulmonary fungal burden was determined on days 7 (**A**) and 14 (**B**) post-infection. Brain fungal burden was determined at day 14 post-infection (**C**). Pulmonary fungal burden at day 14 (**B**) was significantly lower in 5-LO^−/−^ mice than in C57BL/6 mice (*n* = 16 mice). Data are expressed as ±SEM and are cumulative of three experiments utilizing five to six mice per group per timepoint. Significant differences were defined as **P < 0.01.

Earlier studies employed intravenous and intratracheal mouse models of cryptococcosis to show that *Cryptococcus* exploits host 5-LO for penetration across the brain–blood barrier (BBB) (17). To investigate the role of host 5-LO on protective immune responses following pulmonary cryptococcal infection, we sought to exploit a similar model using a *Cryptococcus* species that causes a long-term, chronic infection in C57BL/6 mice (18–20). Mice were given an experimental pulmonary infection with *C. deneoforman*s strain 52D, and pulmonary fungal burden was determined at days 7 and 14 post-infection, and brain fungal burden was quantified at day 14 post-infection. We did not observe a significant difference in pulmonary fungal burden at day 7 post-infection in 5-LO^−/−^ mice compared to C57BL/6 mice (Fig. 1A). However, 5-LO^−/−^ mice showed significantly less pulmonary fungal burden compared to C57BL/6 mice by day 14 post-infection (*P <* 0.005) (Fig. 1B). We observed no overall significant difference in fungal burden within brain tissues of 5-LO^−/−^ mice compared to C57BL/6 infected mice. Nonetheless, we observed that a greater number of 5-LO^−/−^ mice, 14 out of 16 (87.5%) mice, had brain tissues with detectable yeast compared to seven out of 16 C57BL/6 mice (43.75%), with yeast detected in their brains at day 14 post-infection (Fig. 1C). Overall, these data suggest no dependence of host 5-LO by *C. deneoformans* for penetration into brain tissues during the observation period in our study.

### 5-Lipoxygenase deficiency does not alter pulmonary leukocyte infiltration during *C. deneoformans* pulmonary infection

**Fig 2:**
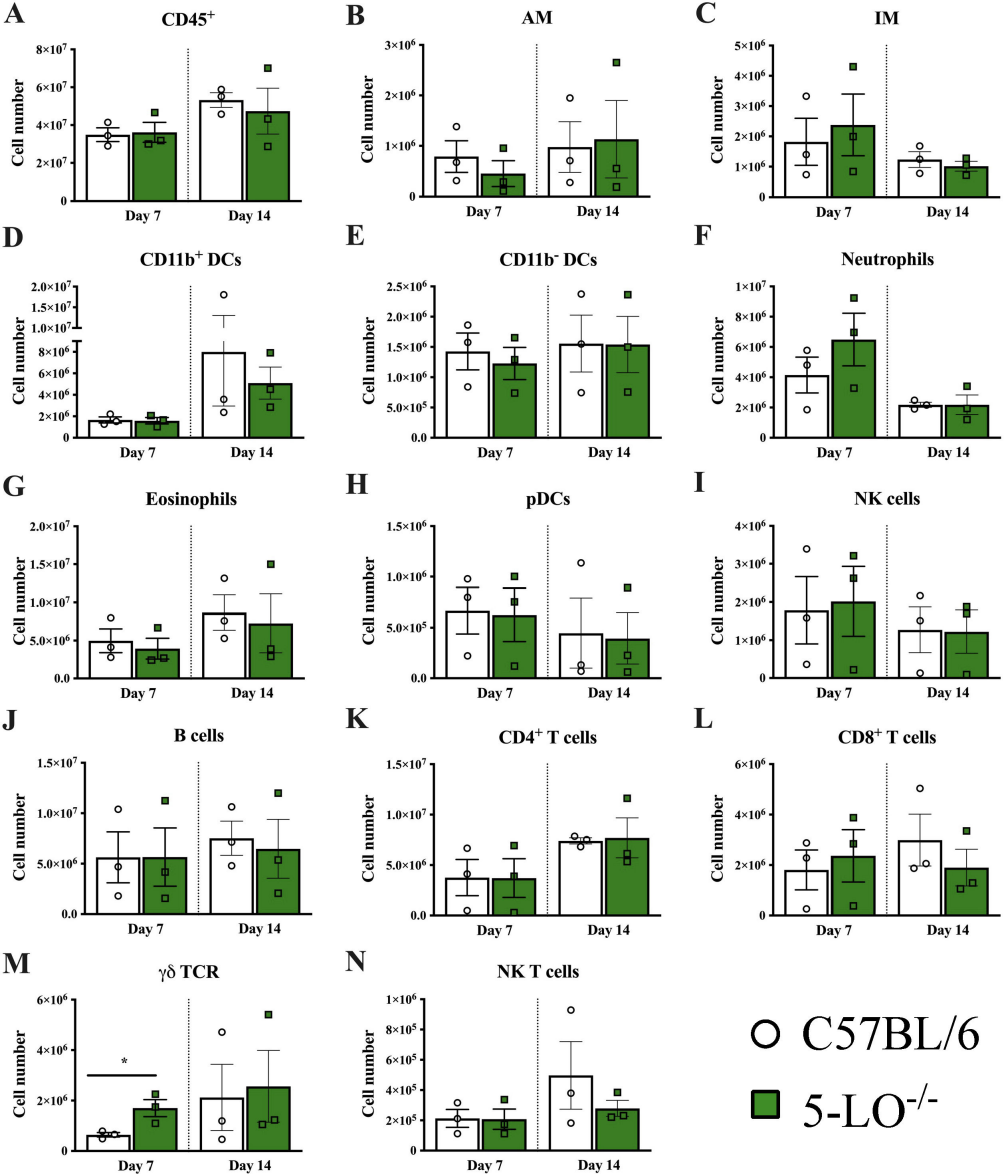
Lack of 5-LO does not impact leukocyte recruitment during early infection with *C. deneoformans*. Total leukocyte infiltration was evaluated at days 7 and 14 post-inoculation in C57BL/6 and 5-LO^−/−^ mice following infection with *C. deneoformans* strain 52D. Lung tissues of C57BL/6J and 5-LO^−/−^ mice were excised, and tissues within each group were combined prior to isolation of leukocytes at each timepoint. Leukocytes were then labeled with anti-CD45 antibodies for total leukocytes (**A**) or labeled with anti-CD45 and antibodies specific for each cell type (**B–N**) and were analyzed by flow cytometry. Data shown are the mean ± SEM of absolute cell numbers from three independent experiments performed using five mice per group per timepoint per experiment. A significant difference is defined as *P < 0.05*.*

LTB_4_ potentiates leukocyte recruitment to inflammatory sites during infection (21). Therefore, we determined the effect of host 5-LO deficiency on recruitment of leukocytes during pulmonary cryptococcosis. Infiltration of leukocytes to the lungs of 5-LO^−/−^ mice and C57BL/6 mice was evaluated at days 7 and 14 post-infection with *C. deneoformans* (Fig. S1). We observed no statistically significant differences in total CD45^+^ leukocyte infiltrates in the lungs of C57BL/6 and 5-LO^−/−^ mice on days 7 or 14 post-infection (Fig. 2A). Additionally, we observed no significant differences in myeloid cells, including alveolar macrophages (AM), interstitial macrophages (IM), dendritic cells, eosinophils, and neutrophils at both days 7 and 14 post-infection (Fig. 2B through H). A similar trend was observed in the adaptive immune response. We did not observe a significant difference in T cell responses between C57BL/6 and 5-LO^−/−^ mice at days 7 and 14 post-infection (Fig. 2J through N), except that γδ T cells were significantly increased at day 7 post-infection in 5-LO^−/−^ mice compared to C57BL/6 mice (Fig. 2M).

We then determined the potential impact of host 5-LO deficiency on the antifungal activity of macrophages against *C. deneoformans* during infection, as previously reported. We observed no difference in the antifungal activity of macrophages extracted from C57BL/6 and 5-LO^−/−^ mice at days 7 and 14 post-infection immediately following isolation and after 24 hours of culture (Fig. S1). Altogether, these data suggest that host 5-LO is not required to recruit leukocytes to the lungs during pulmonary infection with *C. deneoformans*.

Since leukotrienes are lipid mediators that can exhibit potent immunomodulatory properties (21), we analyzed cytokine and chemokine levels in 5-LO^−/−^ mice, compared to C57BL/6 mice, during infection. We observed significant increases in proinflammatory cytokines IL-6 and IL-1β in the lungs of 5-LO^−/−^ mice, compared to levels in C57BL/6 mice, at day 7 post-infection (Table 1). Similarly, we observed significant increases in IL-17, G-CSF, CXCL1, CCL2, CCL3, CCL4, and CCL11 production in 5-LO^−/−^ mice, compared to C57BL/6 mice, at day 7 post-infection (Table 1). However, no significant differences in cytokine and chemokine levels were observed between 5-LO^−/−^ and C57BL/6 mice at day 14 post-infection (Table 1).

**TABLE 1:** Cytokine profile during pulmonary infection by *C*. de*neoformans* in 5-LO-deficient mice^*a*^

Cytokine	Day 7		Day 14	
	WT	5-LO KO	WT	5-LO KO
IFN-γ	41.38 ± 18.83	47.02 ± 5.158	67.06 ± 7.312	60.07 ± 8.071
IL-2	24.34 ± 6.096	29.67 ± 4.426	30.86 ± 2.876	24.18 ± 3.338
IL-12p40	211.3 ± 52.02	386.8 ± 71.43	158.4 ± 16.56	157.7 ± 12.81
IL-12p70	70.15 ± 40.38	87.02 ± 11.80	174.6 ± 41.24	141.3 ± 33.47
TNF-α	55.42 ± 32.91	55.17 ± 13.80	132.5 ± 23.59	94.86 ± 22.82
IL-4	4.789 ± 1.389	6.042 ± 1.699	32.93 ± 4.012	9.524 ± 1.893****
IL-5	4.271 ± 1.560	6.597 ± 0.9932	10.15 ± 1.695	9.330 ± 2.187
IL-10	12.89 ± 6.966	13.42 ± 3.313	35.64 ± 4.497	34.79 ± 6.028
IL-13	199.2 ± 89.95	126.8 ± 32.73	232.8 ± 44.79	194.5 ± 58.69
IL-1α	75.89 ± 8.871	108.6 ± 20.50	48.82 ± 4.527	45.72 ± 5.105
IL-1β	9.447 ± 2.524	18.56 ± 1.995*	24.41 ± 4.101	18.67 ± 2.713
IL-6	20.91 ± 4.700	52.99 ± 11.31*	12.66 ± 1.620	9.197 ± 1.263
IL-17	3.684 ± 0.5645	16.25 ± 5.589*	6.527 ± 0.4774	8.503 ± 1.230
G-CSF	62.30 ± 17.95	357.0 ± 70.87***	12.37 ± 1.287	30.55 ± 9.785
GM-CSF	2.875 ± 0.9885	3.793 ± 1.347	4.026 ± 1.620	3.972 ± 1.411
CXCL1 (KC)	51.83 ± 15.03	176.7 ± 38.52**	37.82 ± 6.857	32.67 ± 5.335
CCL2 (MCP-1)	306.7 ± 89.03	749.7 ± 173.4*	139.6 ± 21.95	117.2 ± 18.89
CCL3 (MIP-1α)	97.85 ± 27.11	303.3 ± 76.34*	101.8 ± 19.39	89.19 ± 18.27
CCL4 (MIP-1β)	40.50 ± 6.767	80.35 ± 13.73*	44.88 ± 4.509	51.53 ± 10.25
CCL5 (RANTES)	897.7 ± 227.7	1,005 ± 194.4	471.4 ± 80.08	584.6 ± 74.87
CCL11 (eotaxin)	150.6 ± 21.08	274.1 ± 30.52**	286.4 ± 22.80	310.8 ± 51.62

^*a*^ Cytokines were measured on days 7 and 14 post-infection from lung supernatants of C57BL/6 and 5-LO^−/−^ mice infected with 10^4^ CFU of *C. deneoformans* 52D per mouse. Data shown are expressed as mean ± SEM and are cumulative of two experiments utilizing five mice per group (male and female mice). Bolded values indicate a significant increase in 5-LO^−/−^ mice compared to C57BL/6 mice. Italicized values indicate a significant decrease in 5-LO^−/−^ mice compared to C57BL/6 mice. Significant differences were defined as *P < 0.05, **P < 0.01, and ****P < 0.0001.

### 5-Lipoxygenase-deficient mice demonstrate reduced mortality against *C. deneoformans*

**Fig 3:**
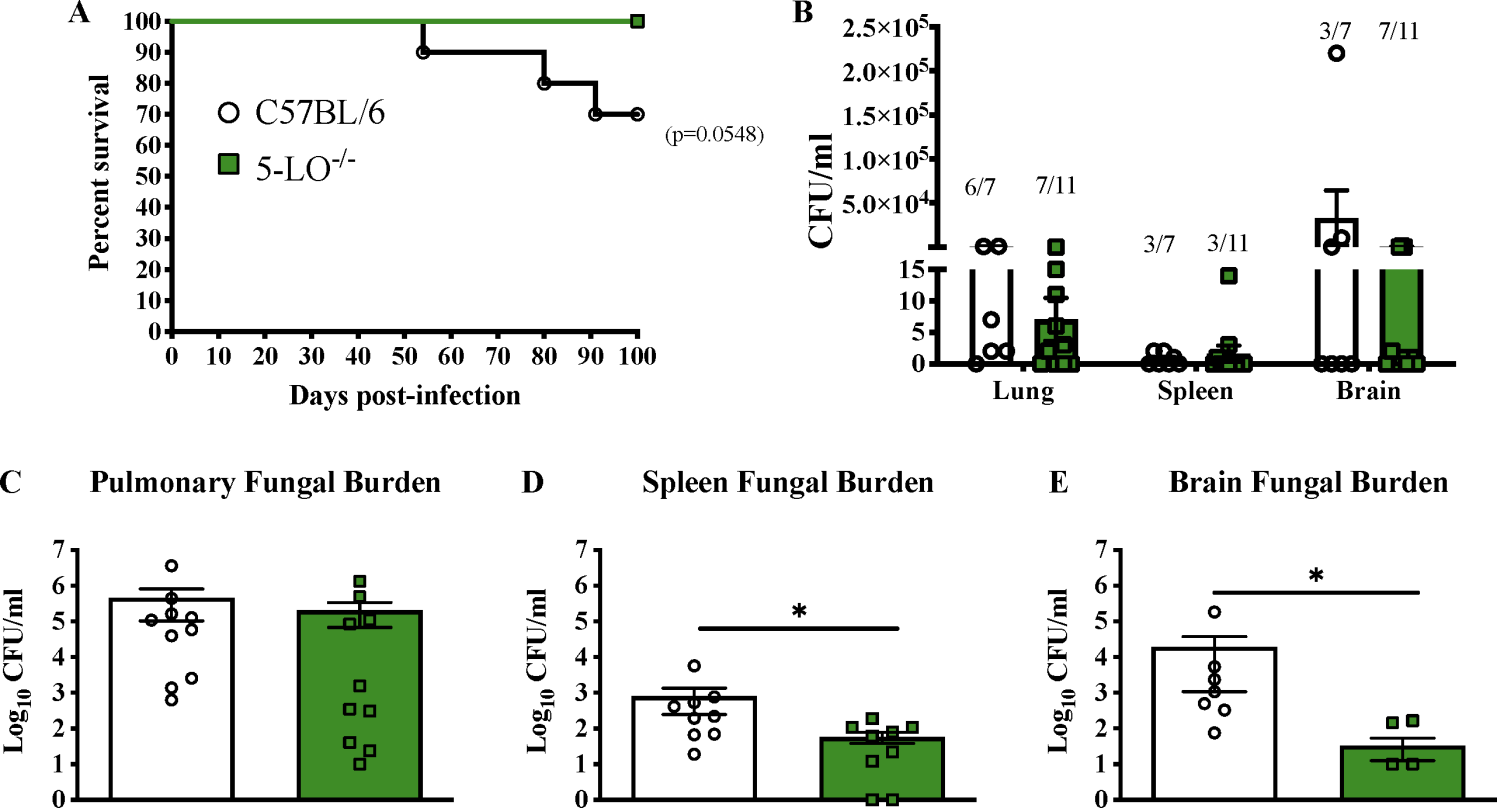
Blockade of host 5-LO prolongs survival and reduces morbidity associated with meningoencephalitis during *C. deneoformans* infection. (A–E) C57BL/6 and 5-LO^−/−^ mice were infected via intranasal inhalation with *C. deneoformans* strain 52D at 10^4^ CFU per mouse. Mice (*n* = 11; male and female) were observed up to day 100 for survival, (**A**) and fungal burden (**B**) in lung, spleen, and brain tissues was determined upon termination of the survival experiment at day 100 post-infection. Fungal burden was determined at day 35 post-infection in the lung (**C**), spleen (**D**), and brain (**E**) (*n* = 10 male and female). Data shown are the mean ± SEM of colony-forming units (CFU/mL) from three independent experiments performed using three to four mice per group (**C–E**). Significant differences were defined as *P < 0.05*.*

To determine the impact of host 5-LO on mortality during pulmonary cryptococcosis, 5-LO-deficient and C57BL/6 mice were given an experimental pulmonary infection with *C. deneoforman*s strain 52D, and survival (morbidity) was monitored for up to 100 days post-infection. 5-LO^−/−^ mice infected with *C. deneoformans* strain 52D remained healthy upon termination of the experiment at day 100 post-inoculation. In contrast, C57BL/6 mice started showing classical signs of cryptococcal meningoencephalitis (enlarged head, weight loss, and imbalance) beginning at day 50 post-infection (Fig. 3A; Fig. S2) and demonstrated a 30% mortality rate by day 100 post-infection (*P* = 0.0548). 5-LO^−/−^ and C57BL/6 mice exhibited relatively low fungal burden in spleen tissues at day 100 post-infection (Fig. 3B). Moreover, while yeasts were detected in lung and brain tissues of C57BL/6 and 5-LO^−/−^ mice, no significant differences in fungal burden were observed in lung and brain tissues of surviving C57BL/6 mice compared to 5-LO^−/−^ mice at day 100 post-infection (Fig. 3B). Importantly, although equivalent levels of fungal burden were detected in the brains of 5-LO^−/−^ and C57BL/6 mice at day 100 post-infection, 5-LO^−/−^ mice did not show any signs associated with meningoencephalitis (Fig. S2).

### Reduced levels of cytokines associated with exacerbation of C-IRIS and less leukocyte cellular infiltrates are observed in brain tissues of 5-lipoxygenase-deficient mice infected with C*. deneoformans*

Although we did not observe any impact on the pulmonary leukocyte infiltration to lung tissues in response to pulmonary cryptococcal infection in 5-LO^−/−^ mice, we noted that 5-LO^−/−^ mice exhibited a reduction in mortality and displayed no outward manifestations of the disease during infection with *C. deneoformans* (Fig. 3A and B). Considering that disease progression is delayed and signs of cryptococcal meningoencephalitis manifest later in C57BL/6 mice following an experimental pulmonary infection with *C. deneoformans,* we aimed to evaluate host responses in brain tissues on day 35 post-infection. Although the pulmonary fungal burden in 5-LO^−/−^ mice was significantly decreased compared to C57BL/6 mice on day 14 post-infection (Fig. 1B), the data in our study demonstrated no differences in pulmonary fungal burden at day 35 post-infection (Fig. 3C). Regarding the dissemination to the brain, or splenic tissues, we found a significant decrease in the fungal burden of 5-LO^−/−^ mice, compared to C57BL/6 mice, at day 35 post-infection (Fig. 3D and E). Nevertheless, 5-LO KO mice did not appear to show any signs associated with meningoencephalitis, resulting in significantly less morbidity and prolonged survival.

**Fig 4:**
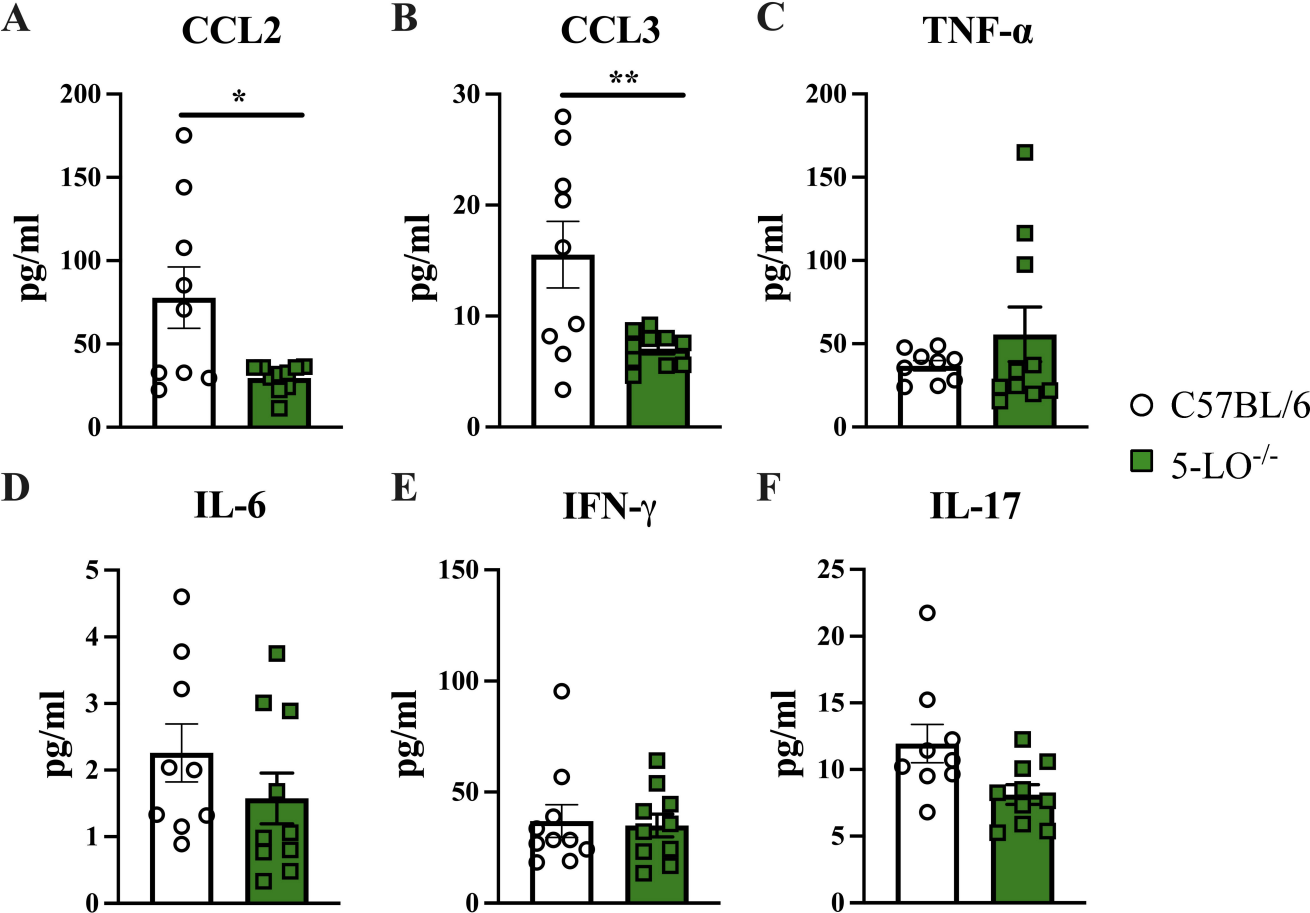
Chemokines associated with high mortality in C-IRIS patients are reduced in 5-LO-deficient mice during *C. deneoformans* infection. Cytokines within brain homogenates of C57BL/6 and 5-LO^−/−^ mice infected with *C. deneoformans* 52D were measured on day 35 post-infection. Data shown are expressed as mean ± SEM and are cumulative of three experiments utilizing three to four mice per group. CCL2 and CCL3 were significantly lower in brain homogenates of 5-LO^−/−^ mice compared to C57BL/6 mice. Significant differences were defined as *P < 0.05.

Next, we compared cytokine levels in homogenates prepared from brain tissues recovered from 5-LO^−/−^ mice and C57BL/6 mice at day 35 post-infection. 5-LO^−/−^ mice showed a significant decrease in the production of CCL2 (*P* = 0.0141) and CCL3 (*P* = 0.0092) chemokines (Fig. 4A and B) compared to C57BL/6 mice. Both chemokines, CCL2 and CCL3, are associated with detrimental outcomes in patients with C-IRIS (22). No differences in the production of TNF-α, IL-6, IFN-γ, and IL-17, cytokines associated with a positive outcome in IRIS patients (22, 23), were detected in brain homogenates of 5-LO^−/−^ mice compared to C57BL/6 mice at day 35 post-infection (Fig. 4C through F).

**Fig 5:**
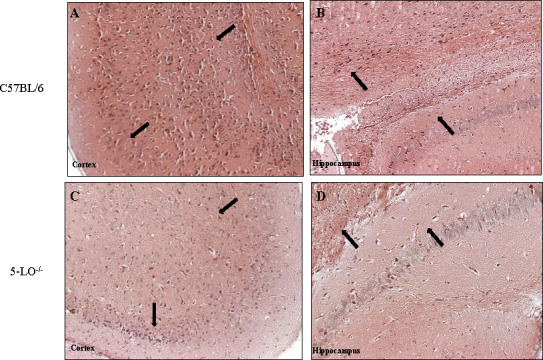
5-LO deficiency results in reduced myeloid cell infiltration into brain tissues during *C. deneoformans* infection. C57BL/6 and 5-LO^−/−^ mice were infected with 10^4^ CFU of *C. deneoformans* strain 52D via intranasal inhalation, and their brains were excised on day 35 post-infection. Sections of brain tissues taken from C57BL/6 (**A and B**) and 5-LO^−/−^ (**C and D**) infected mice were stained with hematoxylin and eosin (H&E), and images were taken at 20× objective power. Images are representative of images derived from two experiments using three mice per group.

To further evaluate the role of 5-LO in regulating inflammation, we evaluated cell infiltrates within brain tissues of mice infected with C*. deneoformans* strain 52D. Histopathology of brain tissues taken at day 35 post-infection showed that C57BL/6 mice appeared to have more leukocyte infiltrates in the cortex and hippocampus (Fig. 5A and B) compared to 5-LO^−/−^ mice (Fig. 5C and D). These results suggest that abrogation of host 5-LO results in a reduction in inflammatory responses in brain tissues during *C. deneoformans* infection.

### Inhibition of host 5-lipoxygenase results in a significant delay in signs associated with disease and mortality associated with *Cryptococcus* meningoencephalitis

**Fig 6:**
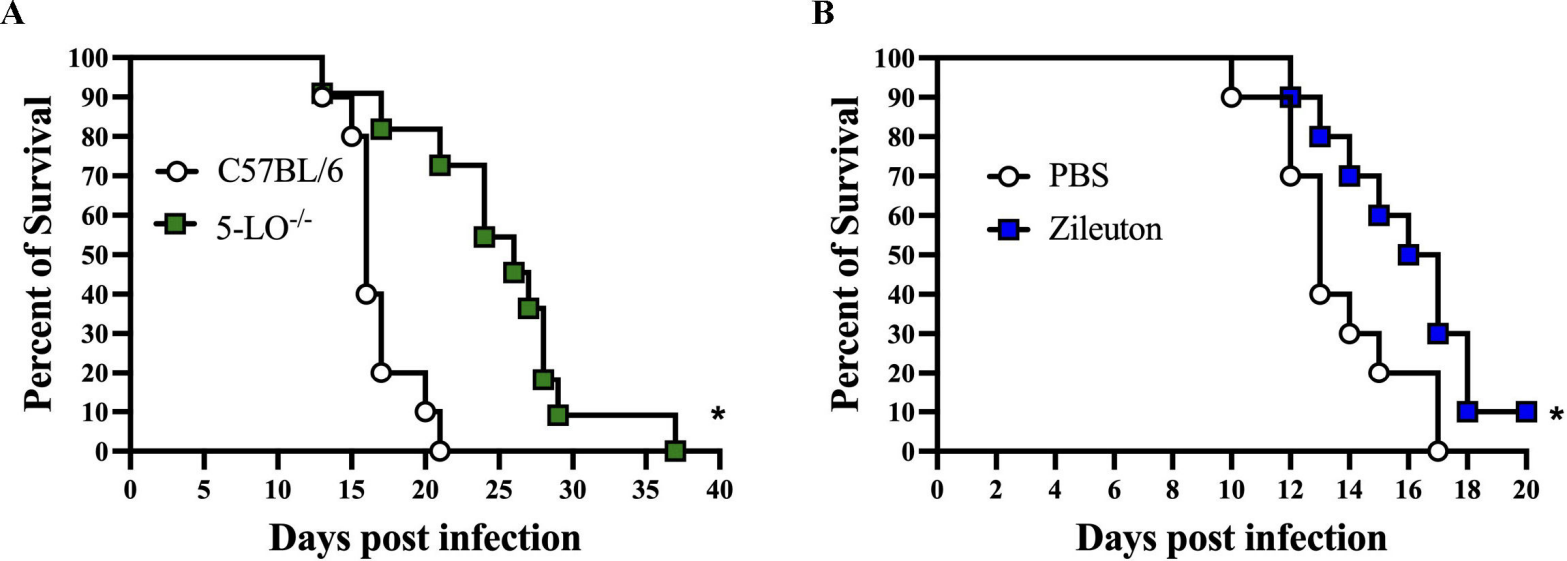
5-LO deficiency results in reduced mortality following intravenous infection with *C. deneoformans*. C57BL/6 and 5-LO^−/−^ mice were infected intravenously (iv) with 10^4^ CFU of *C. deneoformans* strain 52D, and mortality was monitored daily for survival up to day 40 post-infection (*n* = 10 mice). (**A**) C57BL/6 mice were infected iv with *C. deneoformans* strain 52D at 10^4^ CFU per mouse and treated with zileuton (70mg/kg) or sterile PBS three times daily, and survival was monitored daily (*n* = 19). (**B**) Significant differences were defined as *P < 0.05; **P < 0.01.

The observation of less cell infiltration to brain tissues of 5-LO^−/−^ mice in the histopathology and decreased levels of CCL2 and CCL3 levels in brain homogenates of 5-LO^−/−^, compared to C57BL/6 mice during pulmonary infection with *C. deneoformans,* led us to evaluate the impact of host-derived 5-LO using a systemic mouse model of cryptococcosis. 5-LO^−/−^ and C57BL/6 mice received an intravenous inoculation with *C. deneoformans* and were monitored daily for mortality. Figure 6A shows that C57BL/6 mice succumbed to disease significantly earlier than 5-LO^−/−^ mice (median survival time of 16 and 24 days in C57BL/6 and 5-LO^−/−^ mice, respectively). 5-LO^−/−^ and C57BL/6 infected mice displayed signs associated with meningoencephalitis at the time of sacrifice (enlarged head, weight loss, and imbalance; Fig. S3); however, the delay in neurological deterioration suggests that inhibition of 5-LO synthesis results in a delay in the development of the inflammation and pathology associated with cryptococcal meningoencephalitis.

Based on the result of the systemic infection mouse model in the 5-LO^−/−^ mice, we decided to evaluate the putative impact of 5-LO inhibitors as a potential therapy for reducing brain inflammation and subsequent mortality associated with *Cryptococcus* meningoencephalitis. To do this, C57BL/6 mice were given an experimental infection with *C. deneoformans* via intravenous inoculation and, 24 hours later, animals were treated with zileuton, an FDA-approved 5-LO inhibitor, at a dosage approved for clinical use in humans or sterile PBS three times a day for the duration of the experiment. Figure 6B shows a significant increase in survival for the mice treated with zileuton compared to mock-treated mice (median survival of 13 and 16.5 days in PBS and zileuton-treated mice, respectively). Taken together, the data suggest that therapies that inhibit host 5-LO synthesis and/or signaling could aid in control of brain inflammation, resulting in reduced mortality during pulmonary and systemic cryptococcosis.

## DISCUSSION

The pathological outcome of the host*–Cryptococcus* interaction can result from damage to the host caused by the microorganism and/or the host response to infection (24). While most environmental exposures to *Cryptococcus* species result in no outward signs of disease, it is clear that the immune status of the host can have a significant impact on disease outcomes (25). Leukotrienes (LTs) are part of a group of lipids made by the oxidation of polyunsaturated fatty acids (4, 5). 5-Lipoxygenase (5-LO) metabolizes arachidonic acid into LTs, which play an essential role in acute and chronic inflammation and allergic disease (6). One of the most common LTs, LTB_4_, is synthesized by phagocytes during inflammation or infection and stimulates the migration of leukocytes to sites of inflammation. LTB4 is also capable of enhancing phagocytosis and phagocyte antimicrobial activity (7–10). Previous studies aimed to elucidate mechanisms by which cysteinyl leukotrienes contribute to *C. neoformans* penetration across the brain–blood barrier (BBB). This study noted a significant reduction in *C. neoformans* penetration into the brains of 5-LO^−/−^ mice following intravenous inoculation (17). Additionally, Zhu and colleagues identified that pharmacological inhibition of 5-LO using the selective 5-LO inhibitor zileuton inhibits *C. neoformans* transversal in the *in vitro* BBB model (17). These previous studies did not evaluate the efficacy of 5-LO inhibition on host responses during infection or *Cryptococcus* penetration across the BBB *in vivo* post-day 7 of infection. C57BL/6 mice infected with *C. deneoformans* strain 52D develop a chronic lung infection (18–20), followed by dissemination to the brain, showing signs consistent with cryptococcal meningoencephalitis (enlarged head, weight loss, and imbalance). Consequently, we postulated that employing this *Cryptococcus* strain in our studies would allow us to evaluate the long-term impact of host 5-LO on host responses during cryptococcosis.

5-LO-deficient mice given an experimental pulmonary infection with *C. deneoforman*s strain 52D were observed to have significantly less pulmonary fungal burden compared to WT mice on day 14 post-infection. However, we observed no significant differences in pulmonary fungal burden between 5-LO^−/−^ and C57BL/6 infected mice at day 35 onward, suggesting that any impact that 5-LO deficiency may have on host responses to cryptococcosis in the lung may be short-lived. We also observed no significant differences in the antimicrobial activity of macrophages extracted on days 7 and 14 from 5-LO^−/−^ and C57BL/6 mice infected with *C. deneoformans* at the time of isolation and following 24 hours of culture (Fig. S1). Additionally, while we observed significant increases in several proinflammatory cytokines and chemokines in the lungs of 5-LO^−/−^ mice, compared to levels in C57BL/6 mice, at day 7 post-infection, no differences were observed at day 14 post-infection. LTB_4_ stimulates inflammatory pathways and increases the production of proinflammatory cytokines and chemokines. LTB_4_, through BLT1 and BLT2, enhances NF-κB DNA binding and the production of proinflammatory cytokines and chemokines (IL-6, CCL2, and TNF-α). Blockage of LTB_4_ receptors inhibits NF-κB DNA binding and inhibits mRNA expression of proinflammatory cytokines (26–28). During cryptococcal infection, the lack of host 5-LO had the opposite effect on the production of proinflammatory cytokines like IL-6, G-CSF, and CCL2, suggesting that *Cryptococcus* can stimulate the production of these cytokines independently of the host LTB_4_.

Interestingly, although one of the main functions attributed to LTB_4_ is the recruitment of myeloid to sites of inflammation (10, 29), we observed no differences in the recruitment of myeloid or T cells to lung tissues of 5-LO KO mice compared to C57BL/6 mice post-day 7 post-infection or macrophage antimicrobial activity. Experimental murine models of *P. brasiliensis* and *H. capsulatum* infection showed that 5-LO deficiency results in decreased neutrophil recruitment (8, 15, 16). LTB_4_ was demonstrated to be required for the recruitment of both neutrophils and eosinophils in mice with pulmonary aspergillosis (16), and blockage of LTB_4_ results in decreased uptake in *Klebsiella pneumoniae* and *Leishmania* infections (11, 30). Nonetheless, host 5-LO does not appear to be critical for macrophage antimicrobial activity or required to recruit leukocytes to the lungs during infection with *C. deneoformans*.

Cryptococcal meningoencephalitis (CM) is the most common disseminated fungal disease in AIDS patients (2, 31). Studies suggest that the specific factors driving the deleterious inflammatory responses leading to immune reconstitution inflammatory syndrome (IRIS) in HIV-associated cryptococcal meningoencephalitis patients may be associated with particular chemokine responses that promote myeloid cell infiltration into the CNS (22, 23). Jarvis et al. demonstrated that increased baseline levels of interleukin-6 (IL-6), gamma interferon (IFN-γ), IL-8, IL-10, IL-17, RANTES/CLL5, and tumor necrosis factor alpha (TNF-α) in the cerebrospinal fluid (CSF) of HIV^+^ patients were associated with increased macrophage activation, more rapid clearance of cryptococci from CSF, and survival. In contrast, high baseline levels of chemokines CCL2, CCL3, and granulocyte–macrophage colony-stimulating factor (GM-CSF), chemokines that promote monocyte and neutrophil recruitment into the CNS, in the CSF are associated with early mortality and development of IRIS. In the current study, we observed significantly less levels of chemokines associated with detrimental outcomes in patients with IRIS (CCL2 and CCL3) and similar levels of cytokines associated with a positive outcome in IRIS patients (TNF-α, IL-6, IFN-γ, and IL-17) in brain tissues of 5-LO^−/−^ mice compared to WT mice. The results of these studies also align with previous findings by Chang et al., which demonstrate that increased expression of CCL2 and CCL3 in the CSF is associated with subsequent development of C-IRIS (32). Therefore, treatment with 5-LO inhibitors could help minimize inflammation in the CNS, which leads to detrimental outcomes resulting from cryptococcal meningoencephalitis.

Host 5-LO appears to be pivotal in fungal infections caused by *Paracoccidioides brasiliensis*, *Histoplasma capsulatum*, and *Aspergillus fumigatus*, where lack of host 5-LO results in increased mortality (8, 13, 15, 16). Previous studies suggested that *Cryptococcus* exploits host 5-LO for transmigration across the BBB (17). In this study, we observed no difference in fungal burden within brain tissues of 5-LO^−/−^ and WT-infected mice assayed at several timepoints (i.e., days 14, 35, and 100) during the study. While WT and 5-LO^−/−^ mice had similar levels of cryptococci within brain tissues upon termination of the survival study, 5-LO^−/−^ mice given an experimental pulmonary cryptococcal infection did not show any signs associated with cryptococcus meningoencephalitis. In contrast, WT mice began showing classic signs of *Cryptococcus* meningoencephalitis beginning at day 50 post-pulmonary infection and demonstrated a 70% survival rate by day 100 post-infection. The previous study evaluated the impact of host 5-LO on transversal across the BBB using in *vitro* and in *vivo* models employing *Cryptococcus* serotype A, B, and D strains and observed similar findings, suggesting that the difference between our results and those in the previous study is not *Cryptococcus* strain-dependent. However, the previous study evaluated the impact of host 5-LO on dissemination of *Cryptococcus* to brain tissues 24 hours and 7 days post-intravenous and -intratracheal, respectively, inoculation. In contrast, this study elected to make such determinations over a significantly longer observation period (up to 100 days post-inoculation). Thus, the potential roles of host 5-LO in the facilitation of *Cryptococcus* trafficking across the BBB and/or the modulation of host immune responses during infection may have differing impacts on disease outcomes as the disease progresses. These results suggest that 5-LO deficiency does not have a significant role in the recruitment of leukocytes to pulmonary tissues of mice infected with *Cryptococcus*, as we did not observe any difference in leukocyte trafficking to the lungs in mice lacking 5-LO compared to WT mice. However, 5-LO deficiency was associated with reduced brain inflammation. Thus, the overall benefit of inhibiting host 5-LO signaling and leukotriene production and/or uptake (via leukotriene receptor antagonists) in this disease context may be the inhibition of overexuberant inflammatory responses that result in disease pathology associated with *Cryptococcus* infection of the CNS. 5-LO is expressed in the CNS and is predominantly produced by microglia (33). The highest amount of 5-LO in the brain is found in the cerebellum compared to the cortex and hippocampus (34). Levels of cysteinyl leukotrienes (CysLTs), LTC_4_, LTD_4_, and LTB_4_ increase in brain injuries (e.g., cerebral ischemia, brain trauma, and tumors), and the CystLT1 receptor increases in neuron and glial cells after trauma or tumor (35). HIV^+^ patients have elevated amounts of LTB_4_ in the CSF (36). Moreover, HIV^+^ patients with *Toxoplasm*a encephalitis have low levels of LTB_4_ and LTC_4_ in the CSF compared to those with encephalitis (37), suggesting that release of LTs is suppressed in the CSF during *T. gondii* infection. These findings suggest that LTs may play an important role in the inflammatory response in the CNS. Pharmacological inhibitors of 5-LO synthesis or leukotriene receptor antagonists appear to not interfere with the activity of amphotericin B or fluconazole, two antifungal agents typically used to treat cryptococcosis patients (17). Thus, a combination therapy that includes 5-LO inhibitors and antifungal drugs may reduce overexuberant inflammatory responses and reduce the uncontrolled propagation and dissemination of *Cryptococcus* to the CNS.

Altogether, our results show that 5-LO^-/-^ mice exhibit a significant reduction in neurological signs associated with cryptococcus meningoencephalitis. 5-LO^−/−^ mice given an experimental pulmonary infection with *Cryptococcus* showed 100% survival and no signs associated with cryptococcus meningoencephalitis upon conclusion of the study compared to 70% survival of WT-infected mice that displayed clinical signs of cryptococcus meningoencephalitis. 5-LO^−/−^ infected mice exhibited significantly lower levels of chemokines associated with detrimental outcomes of cryptococcus meningoencephalitis in brain tissues and less brain inflammation despite having similar levels of fungal burden in brain tissues compared to WT mice. Our results support the therapeutic potential of host 5-LO inhibition to prevent or mitigate the overexuberant inflammatory responses that result in significant morbidity and mortality associated with *Cryptococcus* meningoencephalitis.

## MATERIALS AND METHODS

### Mice

Male and female 5-lipoxygenase-deficient mice (Alox5^tm1Fun^/J) and wild-type C57BL/6 J mice between 5 and 7 weeks of age were acquired from Jackson Laboratories and housed at The University of Texas at San Antonio Small Animal Laboratory Vivarium or the vivarium at The University of North Texas Health Science Center at Fort Worth. All animal experiments were conducted following NIH guidelines for housing and care of laboratory animals and following all relevant protocols and ethical regulations for animal testing and research approved by the IACUC of the University of Texas at San Antonio and Texas Christian University.

### Strains and media

*Cryptococcus deneoformans* strain 52D (serotype D, mating type α) was recovered from 15% glycerol stocks stored at −80°C and maintained on yeast peptone dextrose (YPD) media agar plates (Becton Dickinson, Sparks, MD). Yeast cells were grown for 16–18 hours at 30°C with shaking in liquid YPD broth, collected by centrifugation, washed three times with sterile phosphate-buffered saline (PBS), and viable yeasts were quantified using trypan blue dye exclusion on a hemocytometer.

### Pulmonary cryptococcal infections and fungal burden

Mice were anesthetized with 2% isoflurane utilizing a rodent anesthesia device (Eagle Eye Anesthesia, Jacksonville, FL) and subsequently infected via the intranasal route with 1 × 10^4^ colony-forming units (CFU) of *C. deneoformans* strain 52D in 30 µL of sterile PBS. The inocula used for the nasal inhalation were verified by quantitative culture on YPD agar. Mice were euthanized on predetermined days by CO_2_ inhalation, followed by cervical dislocation, and tissues were excised. The left lobes of the lung, spleen, and brain were removed and homogenized in 1 mL of sterile PBS, as previously described (38), followed by culture of tenfold dilutions of each homogenate on YPD agar supplemented with chloramphenicol. CFU were enumerated following incubation at 30°C for 48 hours. For survival studies, mice were inoculated as stated above, monitored twice daily, and humanely euthanized if moribund.

### Pulmonary leukocyte isolation

Lungs of C57BL/6J and 5-LO^−/−^ mice (*n* = 5 mice/group) were excised on days 7 and 14 post-inoculation, as previously described (38). Lungs were then enzymatically digested at 37°C for 30 minutes in 10 mL of digestion buffer (RPMI 1640 and 1 mg/mL collagenase type IV [Sigma-Aldrich, St. Louis, MO]) with intermittent (every 10 min) stomacher homogenizations. The digested tissues were then successively filtered through sterile 70- and 40-µm nylon filters (BD Biosciences, San Diego, CA) to enrich for leukocytes. The cells were washed with sterile Hanks’ balanced salt solution (HBSS). Erythrocytes were lysed by incubation in NH_4_Cl buffer (0.859% NH_4_Cl, 0.1% KHCO_3_, 0.0372% Na_2_EDTA [pH 7.4]; Sigma-Aldrich) for 3 minutes on ice, followed by addition of twofold excess of sterile PBS. The cells were then washed in PBS, resuspended in 1 mL PBS, and counted for use in flow cytometry experiments.

### Pulmonary macrophage anti-cryptococcal killing assay

Pulmonary F4/80^+^ macrophages were enriched from mice on days 7 and 14 post-inoculation, as described above, and the viability of phagocytic cells was assessed using trypan blue exclusion by using a hemocytometer. Macrophages were cultured at a density of 5 × 10^5^ cells per well, in triplicate, in a 96-well tissue culture plate in R10 media, as previously described in (38). The initial fungal burden was analyzed by lysing phagocytes using sterile deionized water, followed by serial dilution and plating on YPD agar supplemented with chloramphenicol (Mediatech, Manassas, VA) for 48 ours at 30°C. After 24-h incubation at 37°C, macrophages were lysed with water and also serial dilutions plated on YPD agar to enumerate CFU, as described (39).

### Flow cytometry

The standard methodology was employed for the direct immunofluorescence of pulmonary leukocytes. Briefly, in 96-well U-bottom plates, 100 µL containing 1 × 10^6^ cells in PBS were incubated with yellow Zombie viability dye (1:1,000 dilution, Cat. No. 423104, Biolegend, San Diego, CA) for 15 minutes at room temperature followed by washing in FACS buffer. Cells were then incubated with Fc block (1:500 dilution, Cat. No. 553142, clone 2.4G2, BD Biosciences) diluted in FACS buffer for 5 minutes to block nonspecific binding of antibodies to cellular Fc receptors. Cells (1 × 10^6^ cells) were incubated with fluorochrome-conjugated antibodies (Table 2 ) in various combinations to allow for multi-staining, as follows: leukocytes (CD45^+^), CD4 T cells (CD4^+^/CD3^+^/CD11b^−^/CD45^+^), CD8 T cells (CD8^+^/CD3^+^/CD11b^−^/CD45^+^), B cells (CD19^+^/CD45^+^), polymorphonuclear leukocytes (PMNs; CD11b^+^/Ly6G^+^/CD45^+^), alveolar macrophages (AM; F4/80^+^/CD64^+^/CD24^−^/CD11b^−^/CD45^+^), interstitial macrophages (IM; F4/80^+^/CD64^+^/CD24^−^/CD11b^+^/CD45^+^), CD11b^+^ DCs (CD11c^+^/CD24^+^/CD11b^+^/CD45^+^) CD11b^−^ DCs (CD11c^+^/CD24^+^/CD11b^+^/CD45^+^), eosinophils (SiglecF^+^/CD11b^int^/CD24^+^/CD45^+^), plasmacytoid DCs (pDCs; B220^+^,CD11c^+^/pADC^−^1+/CD45^+^), NK cells (NKp46^+^/CD45^+^), and NK T cells (CD3^+^/NKp46^+^/CD45^+^). The cells were then incubated for 30 min at 4 °C. Cells were washed three times with FACS buffer and fixed in 200 µL of 2% ultrapure formaldehyde (Polysciences, Warrington, PA) diluted in FACS buffer (fixation buffer). Fluorescence minus one (FMO) control or cells incubated with either FACS buffer alone or single fluorochrome-conjugated Abs were used to determine positive staining and spillover/compensation calculations, and background fluorescence was determined with FlowJo v.10 Software (FlowJo, LLC, Ashland, OR). Raw data were collected with a Cell Analyzer LSRII (BD Biosciences) using BD FACSDiva v8.0 software at the Cell Analysis Core of the UTSA or the Flow Cytometry Core at UNTHSC, and compensation and data analyses were performed using FlowJo v.10 Software. Cells were first gated for lymphocytes (SSC-A vs FSC-A) and singlets (FSC-H vs FSC-A). The singlet gate was further analyzed for the uptake of live/dead yellow stain to determine live vs dead cells. Live cells were gated on CD45^+^ cell expression (Fig. S4). For data analyses, 100,000 events (cells) were evaluated from a predominantly leukocyte population identified by back gating from CD45^+^ stained cells. The absolute number of each cell population was determined by multiplying the percentage of each gated population by the total number of CD45^+^ cells.

**TABLE 2:** List of antibodies used in this study for flow cytometry

Antibody	Fluorochrome	Clone	Source	Cat #	Dilutions
Rat anti-mouse CD45	FITC	30-F11	eBio (Invitrogen)	11-0451-81	1:200
Rat anti-mouse CD45	APC	30-F11	eBio (Invitrogen)	17-0451-82	1:200
Rat anti-mouse CD45	BV421	30-F11	BD	563890	1:200
Hamster anti-mouse CD3e	Pe-Cy7	145-2C11	eBio (Invitrogen)	25-0031-81	1:200
Rat anti-mouse CD4	FITC	GK1.5	Biolegend	100406	1:100
Rat anti-mouse CD8a	APC	53-6.7	eBio (Invitrogen)	17-0081-82	1:100
Rat Anti-mouse F4/80	PE	BM8	eBio (Invitrogen)	12-4801-82	1:100
Rat anti-mouse CD24	FITC	M1/69	Biolegend	101806	1:200
Rat anti-mouse CD19	PerCP-Cy5.5	6D5	Biolegend	115533	1:100
Hamster anti-mouse CD11c	PerCP-Cy5.5	N418	Invitrogen	45-0114-82	1:100
Rat anti-mouse/human CD11b	BV421	M1/70	Biolegend	101251	1:200
Rat anti-mouse CD11b	PE	M1/70	BD	557397	1:200
Rat anti-mouse Ly6G	PE	1A8	BD	551461	1:100
Anti-mouse CD64 (FcᵧRI)	Pe-Cy7	X54-5/7.1	Biolegend	1393913	1:100
Rat anti-mouse Siglec-F	BV421	E50-2440	BD	565934	1:100
Rat anti-mpuse pDCA-1 (CD317)	APC	eBio927	Invitrogen	17-3172-80	1:50
Rat anti-mouse CD45R/B220	Pe-Cy7	RA3-6B2	BD	561881	1:100
Rat anti-mouse NKp46 (CD335)	APC	29A1.4	eBio (Invitrogen)	50-3351-80	1:100
Hamster anti-mouse TCR gamma/delta	PE	GL3	eBio (Invitrogen)	12-5711-81	1:100
Zombie yellow Fixable Viability Kit			Biolegend	423104	1:500
Rat anti-mouse CD16/CD32 Fc block		2.4G2	BD	553142	1:500

### Cytokine analysis

Cytokine levels within the lung and brain tissue homogenates were analyzed using the Bio-Plex protein array system (Luminex-based technology, Bio-Rad Laboratories, Hercules, CA). Lung and brain tissues were excised and homogenized in ice-cold sterile PBS (1 mL). An aliquot (50 µL) was taken to quantify the pulmonary fungal burden, and an anti-protease buffer solution (1 mL) containing PBS, protease inhibitors, and 0.05% Triton X-100 was added to the homogenate. Samples were then clarified by centrifugation (2,465 × *g*) for 10 minutes. Supernatants were assayed for the presence of IL-1α, IL-1β, IL-2, IL-4, IL-5, IL-6, IL-10, IL-12(p40), IL-12(p70), IL-13, IL-17a, CXCL1, CCL2, CCL3, CCL4, CCL5, CCL11, IFN-γ, tumor necrosis factor α (TNF-α), granulocyte macrophage-colony stimulating factor (GM-CSF), and monocyte colony-stimulating factor (G-CSF) according to the manufacturer’s instructions.

### Histology

Mice were sacrificed according to the approved protocol. Mice were placed under deep anesthesia with 5% isoflurane in an induction chamber and transcardially perfused with cold saline buffer (1× PBS). Dissected brains were post-fixed overnight in 2% PFA and embedded in parafilm sections. After paraffin embedding, 5-mm sections were cut and stained using hematoxylin and eosin (H&E). Staining was performed by personnel at The University of Texas Health Science Center at San Antonio Histology and Immunohistochemistry Laboratory. Sections were analyzed with light microscopy using a Leica microscope DMI600 B (Leica Microsystems, Wetzlar, Germany).

### Statistical analysis.

Survival data were analyzed using the log-rank test to detect statistically significant differences using GraphPad Prism Version 10.1.1 for Macintosh (GraphPad Software, San Diego, CA). The unpaired Student *t*-test was used to analyze fungal burden, cytokine and chemokine levels, pulmonary cell populations by flow cytometry, and anti-cryptococcal activity of macrophages by Prism (GraphPad Software). Significant differences were defined as *P* < 0.05 (*), *P* < 0.01 (**), *P* < 0.001 (***), or *P* < 0.0001 (****), unless otherwise noted.

## SUPPLEMENTAL MATERIAL

Figure S1 mbio.01483-24-s0001.tiff Deficiency in host 5-LO has no impact on macrophage antifungal activity against *Cryptococcus*.

Figure S2 mbio.01483-24-s0002.jpg Signs of meningoencephalitis in C57BL/6 mice following intranasal infection with *C. deneoformans* 52D.

Figure S3 mbio.01483-24-s0003.jpg Signs of meningoencephalitis in C57BL/6 and 5-LO mice following intravenous infection with *C. deneoformans* 52D.

Figure S4 mbio.01483-24-s0004.tiff Gating strategy for flow cytometry analysis of pulmonary leukocytes.

Legends mbio.01483-24-s0005.docx Supplemental figure legends.

ASM does not own the copyrights to Supplemental Material that may be linked to, or accessed through, an article. The authors have granted ASM a non-exclusive, world-wide license to publish the Supplemental Material files. Please contact the corresponding author directly for reuse.
