# Supplementary figures and images for: Inhibition of host 5-lipoxygenase reduces overexuberant inflammatory responses and mortality associated with Cryptococcus meningoencephalitis
Source: mBio. 2024 Jul 31;15(9):e01483-24. doi: 10.1128/mbio.01483-24 (PMC11389364; doi:10.1128/mbio.01483-24)

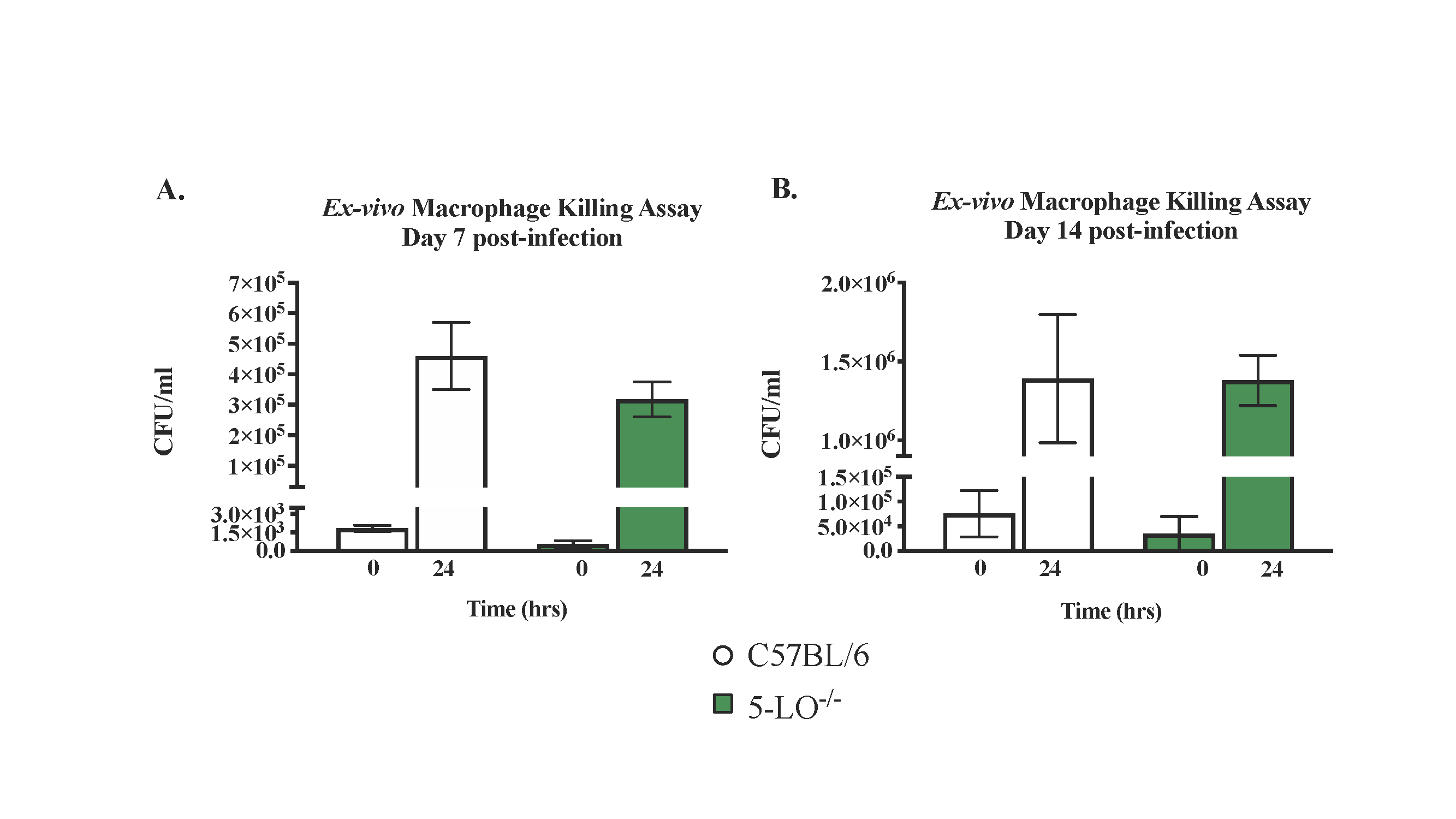

Supplement: Figure S1 — Deficiency in host 5-LO has no impact on macrophage antifungal activity against Cryptococcus. [file mbio.01483-24-s0001.tiff]

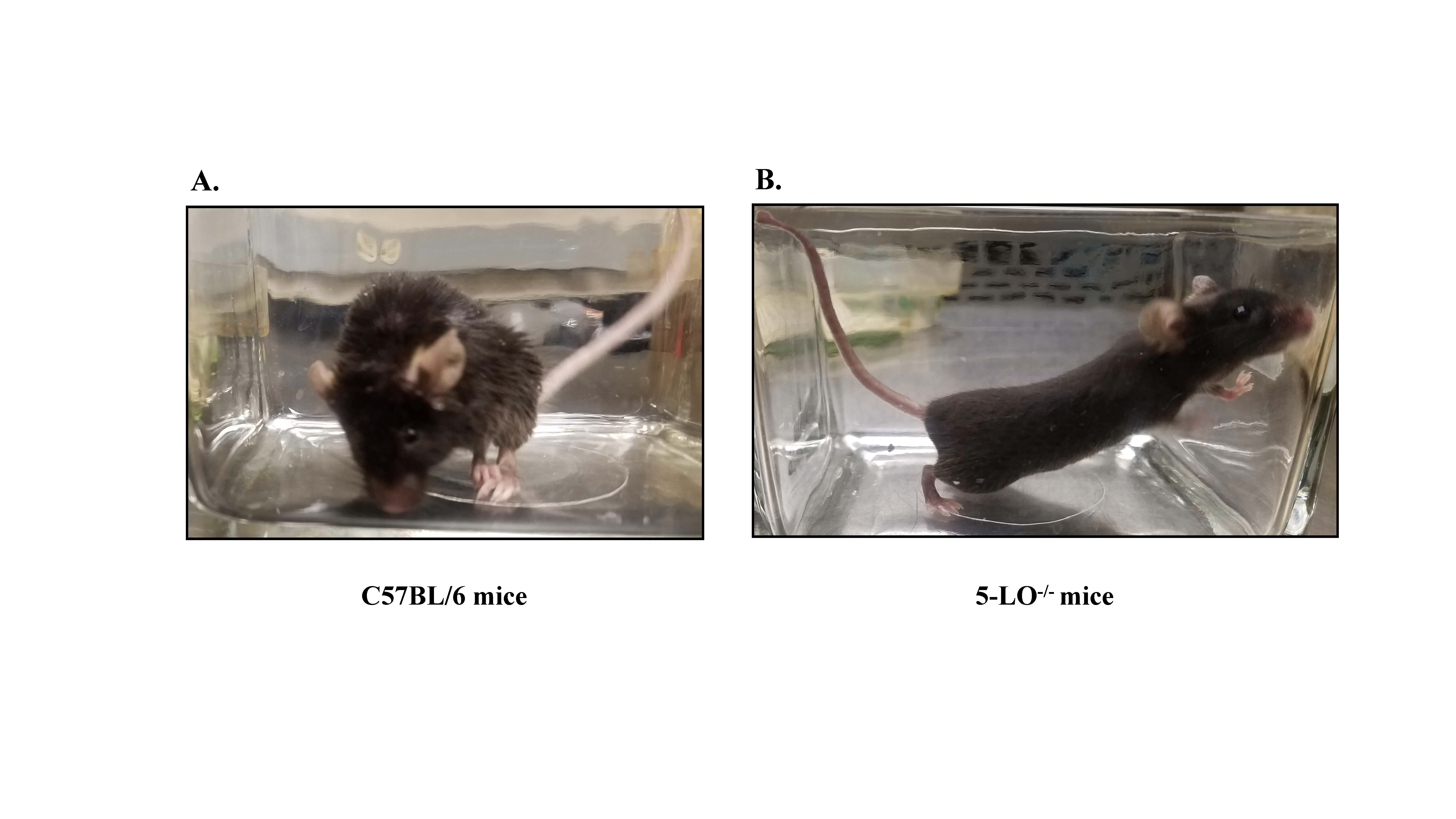

Supplement: Figure S2 — Signs of meningoencephalitis in C57BL/6 mice following intranasal infection with C. deneoformans 52D. [file mbio.01483-24-s0002.jpg]

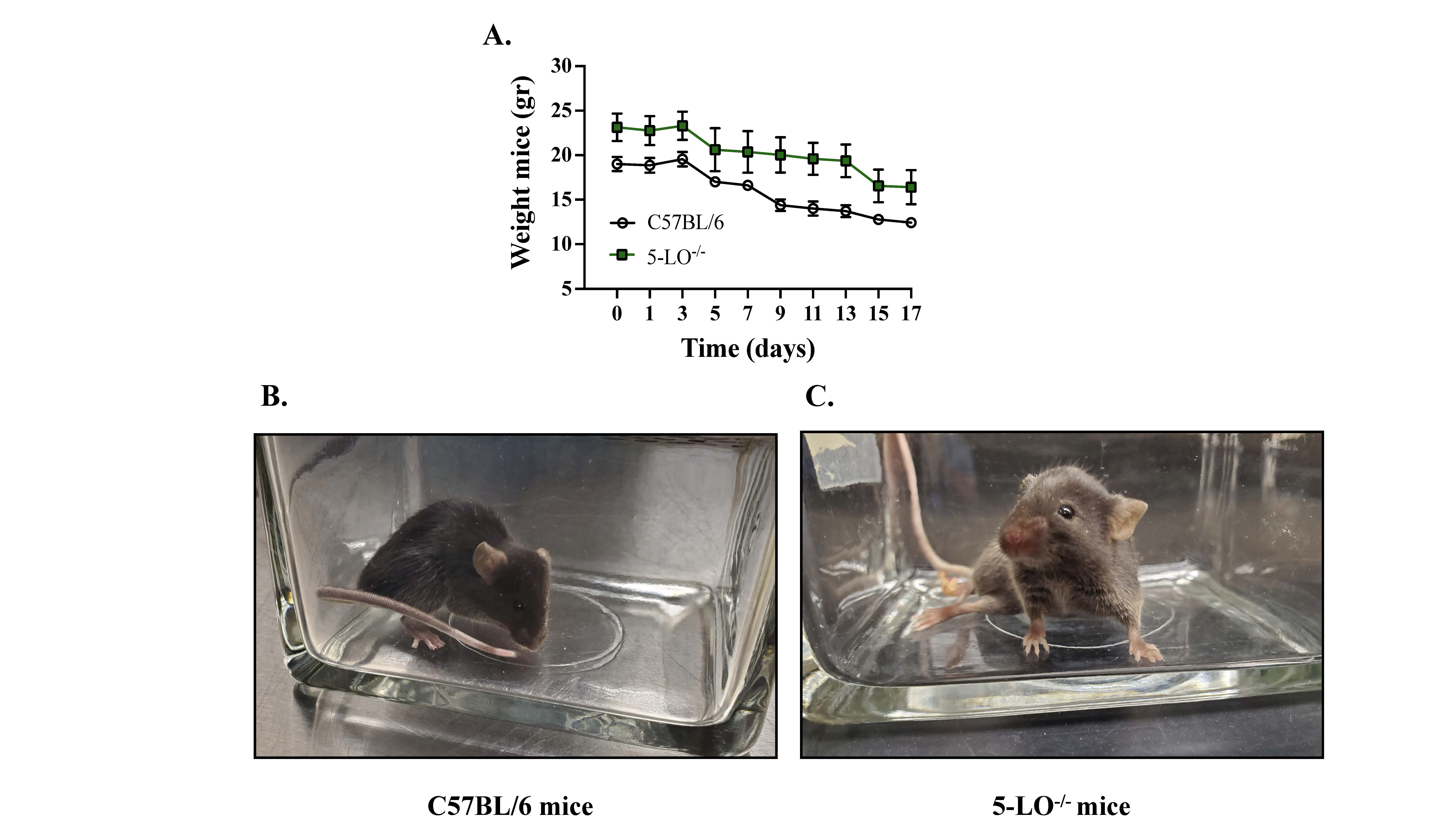

Supplement: Figure S3 — Signs of meningoencephalitis in C57BL/6 and 5-LO mice following intravenous infection with C. deneoformans 52D. [file mbio.01483-24-s0003.jpg]

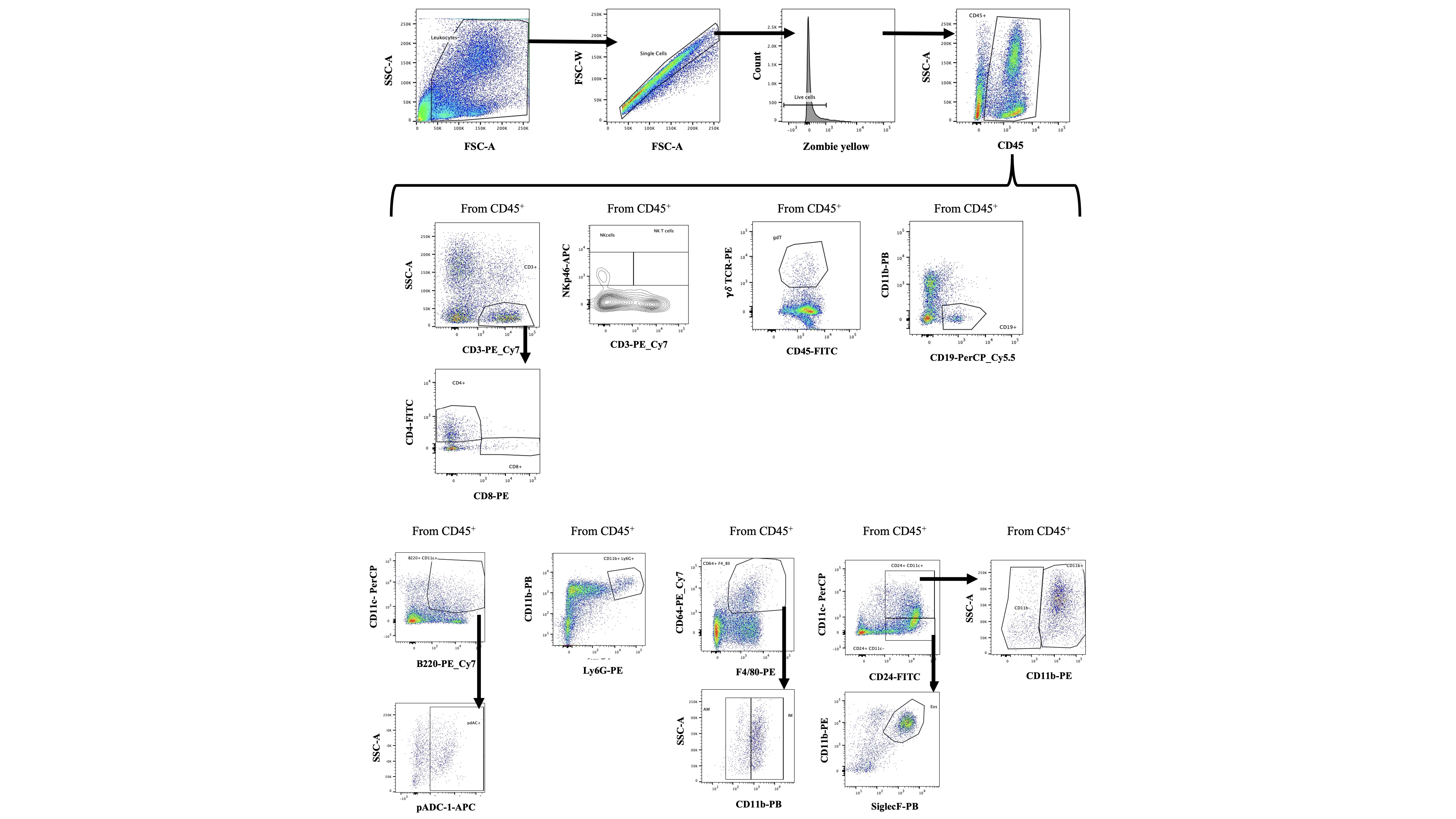

Supplement: Figure S4 — Gating strategy for flow cytometry analysis of pulmonary leukocytes. [file mbio.01483-24-s0004.tiff]
