# Supplementary material for: Inhibition of host 5-lipoxygenase reduces overexuberant inflammatory responses and mortality associated with Cryptococcus meningoencephalitis
Source: mBio. 2024 Jul 31;15(9):e01483-24. doi: 10.1128/mbio.01483-24 (PMC11389364; doi:10.1128/mbio.01483-24)
Supplement: Legends — Supplemental figure legends. [file mbio.01483-24-s0005.docx]

**Supplemental Figure 1. Deficiency in host 5-LO has no impact on macrophage antifungal activity against *Cryptococcus*.** Pulmonary macrophages were isolated from C57BL/6 mice and 5-LO^-/-^ mice on days 7 (A) and 14 (B) post-inoculation and cultured ex vivo for 24 h. The macrophages were subsequently lysed with sterile water to enumerate the intracellular cryptococci. Results are expressed as means ± SEM of two experiments using five mice per group per time point.

**Supplemental Figure 2. Signs of meningoencephalitis in C57BL/6 mice following intranasal infection with *C*. *deneoformans* 52D**. C57BL/6 and 5-LO^-/-^ mice were infected with 10^4^ CFUs of *C. deneoformans* strain 52D per mouse via intranasal inhalation. Mice (n=11; male and female) were observed for up to day 100 post-infection. C57BL/6 (A) and 5-LO^-/-^ (B) mouse photos were taken at day 52 post-infection and are illustrative of how C57BL/6 mice appeared immediately prior to humane sacrifice, compared to 5-LO^-/-^ mice, at time of sacrifice due to signs of C-IRS.

**Supplemental Figure 3. Signs of meningoencephalitis in C57BL/6 and 5-LO mice following intravenous infection with *C*. *deneoformans* 52D**. C57BL/6 and 5-LO^-/-^ mice (n=10 male and female mice/per group) were infected iv with 10^4^ CFUs of *C. deneoformans* strain 52D per mouse and mortality monitored daily up to day 40 post-infection. Weight was monitored every other day after infection (A). C57BL/6 (B) and 5-LO^-/-^ (C) mice

showed classic signs of meningoencephalitis prior sacrifice.

**Supplemental Figure 4. Gating strategy for flow cytometry analysis of pulmonary leukocytes.** C57BL/6 and 5-LO^-/-^ mice were infected with 10^4^ CFUs of *C. deneoformans* strain 52D per mouse via intranasal inhalation. Lung tissues were processed for leukocyte isolation and subsequently prepared for flow cytometry analysis.
